# An online alternative: a qualitative study of virtual abortion values clarification workshops

**DOI:** 10.1080/10872981.2023.2258004

**Published:** 2023-09-18

**Authors:** Margaret Williams, Elise S. Cowley, Taryn M. Valley, Alma Farooque, Zoey Shultz, Amy Godecker, Jacquelyn Askins, Laura Jacques

**Affiliations:** a Dept of Obstetrics and Gynecology, University of Wisconsin-Madison, Madison, USA; b Microbiology Doctoral Training Program, University of Wisconsin-Madison, Madison, USA; c Dept of Bacteriology, University of Wisconsin-Madison, Madison, USA; d Dept of Anthropology, University of Wisconsin-Madison, Madison, USA; e School of Medicine and Public Health, University of Wisconsin-Madison, Madison, USA

**Keywords:** Abortion, medical education, virtual, online, values clarification

## Abstract

**Background:**

Following the U.S. Supreme Court *Dobbs* decision, access to abortion education is increasingly regionally dependent. Participation in values clarification workshops on abortion can improve abortion knowledge and reduce stigma. Traditionally, values clarification workshops occur in person, yet medical education increasingly utilizes online learning. We sought to understand how a virtual platform impacted medical students and Obstetrics and Gynecology (ObGyn) residents’ experience with a values clarification workshop on abortion.

**Methods:**

We conducted values clarification workshops over Zoom with medical students and ObGyn residents at four midwestern teaching hospitals from January 2021-December 2021 during the COVID-19 pandemic. We held semi-structured interviews with participants and facilitators to learn about how the virtual format impacted their experience with the workshop. Four researchers analyzed transcripts using an inductive approach to generate codes then themes.

**Results:**

We interviewed 24 medical students, 13 ObGyn residents, and five workshop facilitators. Participants and facilitators found the virtual platform to have both unique advantages and disadvantages. Four central themes were identified: 1) Screen as a barrier: participants noted obstacles to conversation and intimacy. 2) Emotional safety: participants felt comfortable discussing sensitive topics. 3) Ease of access: participants could access virtual workshops regardless of location. 4) Technology-specific features: Zoom features streamlined aspects of the workshop and allowed for anonymous contributions to discussion.

**Conclusions:**

Our findings suggest that a virtual platform can be a convenient and effective way to deliver values clarification workshops on abortion, and this technology could be leveraged to expand access to this training in areas without trained facilitators.

## Introduction

Teaching on abortion is limited in medical education [1–3], and after the U.S. Supreme Court decision in *Dobbs v. Jackson Women’s Health Organization* overturned the federal right to abortion in June 2022, increasingly regionally dependent [4]. At the time of writing, 15 states have total abortion bans with very limited exceptions (Guttmacher Institute [5], severely restricting training for at least 230 ObGyn residents and an even greater number of medical students (Ryan Residency Training Program [6]. Medical schools and residency programs are now working to fill in the gaps in abortion training for medical trainees [7].

One intervention shown to increase knowledge and support around abortion among participants are Values Clarification and Attitude Transformation (VCAT) Workshops. VCAT workshops have been conducted in a variety of settings around the world [8,9] reaching broad audiences including clinicians, international development workers, and policy makers. In these workshops, participants engage in discussion on abortion-related scenarios, guided by a trained facilitator, and explore their personal and professional beliefs about abortion in an open-minded space [10]. VCAT workshops have traditionally been conducted in-person; however due to COVID-19 in-person learning restrictions and to meet the need for expanded access to abortion education, we adapted a VCAT workshop to a virtual format. Virtual education has been an increasingly utilized and effective method of curriculum delivery [11,12]. The aim of this study was to understand participants’ and facilitators’ experiences participating in the workshop virtually, assessing both strengths and limitations of the virtual format.

## Materials and methods

### Study population

Five facilitators led a total of 29 workshops with medical trainees (medical students and ObGyn residents), of which 26 workshops were with medical students and four with ObGyn residents. We conducted the ‘Four Corners’ portion of a values clarification workshop on abortion [10] at four midwestern teaching hospitals over Zoom. All medical students on their core ObGyn clinical clerkship at three medical schools and all ObGyn residents not on post-call or vacation at four residency programs participated in the workshops virtually from December to January 2021.

### Surveys

Prior to the workshop, participants received an email inviting them to complete a voluntary 23-item Qualtrics survey assessing their attitudes (17 statements) and behavioral intentions (six statements) surrounding abortion and demographic information (Appendix 1). This survey was adapted from a previously published survey evaluating the impact of in-person VCAT workshops with international healthcare workers [9]. Participants received a ten-dollar Amazon gift card for completing the survey. Using the 17 attitudes statements on the survey, we created a summative abortion attitude score ranging from 0 (most negative) to 100 (most positive) for each responder [9].

### Workshop

Prior to the start of each workshop, participants were required to fill out an online Google form containing 12 statements about abortion and select whether they strongly agreed, agreed, strongly disagreed or disagreed with each statement (Appendix 2). Their responses were de-identified, and each participant was emailed a set of responses from one of their colleagues and was asked to participate with those responses during the workshop.

The workshops were facilitated on Zoom by ObGyn faculty at each institution who received a standardized facilitation guide. The facilitator read each of the 12 Google form statements aloud and then opened Zoom polls for each statement, asking participants to reply with their colleague’s de-identified responses. In the traditional Four Corners exercise, each corner of a room is labeled with the four possible responses (Strongly Agree, Agree, Strongly Disagree, Disagree) and participants move to the corner of the room that corresponds with their colleague’s response. Participants are asked to reflect on the visual representation of the variety of beliefs held by their colleagues. Two to three of the statements are used as small group discussions, and people in each corner discuss why someone might hold the belief that is presented on their paper. They then share their thoughts with the large group in a facilitated discussion. In the virtual adaptation moving to the four corners of the room is simulated by responding to a zoom poll and the visual representation of the group’s values is achieved by displaying poll responses to participants. Zoom breakout rooms are used for small group discussions. In both the in-person and virtual workshops, participants work from the beliefs presented on the anonymous form of responses that they receive and not their own beliefs. This encourages empathy building through cognitive flexibility.

### Interviews

**Figure 1.**
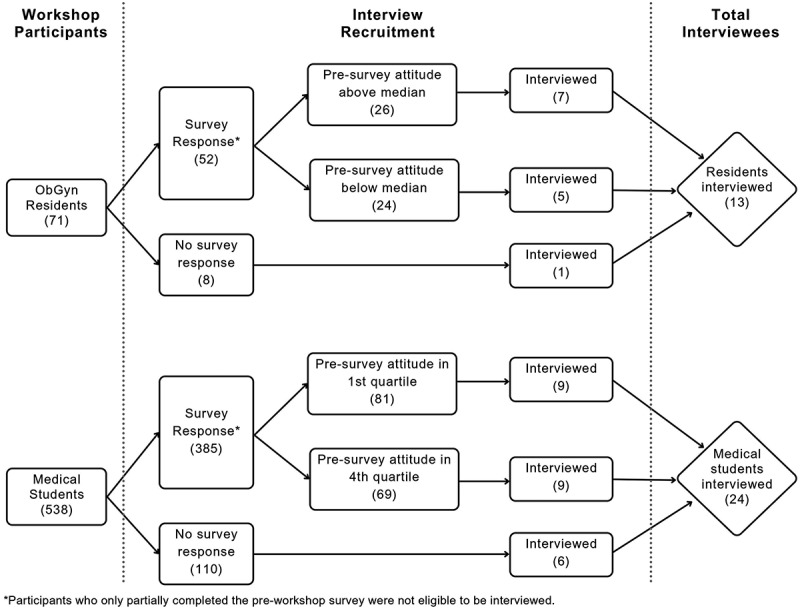
Interview participant recruitment.

After the workshop, a subset of trainees were invited to participate in a semi-structured interview about their experience. To minimize the effect of volunteer bias, the baseline survey abortion attitude scores for participants were sorted from most negative to most positive and grouped into quartiles. We sought to interview three medical students from the lowest and highest attitude quartiles from each school. Of medical students, 72 with attitude scores randomly selected from the highest and lowest quartiles were invited until we reached our target (Figure 1). We also sought to interview three non-responders to the initial survey from each institution. Resident participants were also invited to be interviewed about their experience with a goal of 36 interviews. We emailed four rounds of invitations, or fewer if we reached the goal number of interviewees prior to that. All residents were eventually invited. All facilitators were invited to be interviewed as well. Interview participants received a one-hundred dollar Amazon gift card.

All interviews were conducted virtually on Zoom by one trained interviewer affiliated with the project, but not involved in student or resident evaluation, using a standardized interview guide (Appendix 3), which was created with input from four authors (TMV, ESC, JA, LJ). Interview questions focused on the experience of participating in the Four Corners activity, students’ impressions of their colleagues’ abortion beliefs, implications for future practice, as well as likes, dislikes and surprises about the workshop. Interviews were recorded with participants’ consent and transcribed using Zoom autotranscription followed by final transcription by two authors (TMV, AF).

### Data analysis

We used an inductive qualitative approach to identify common themes in the data [13]. Four qualitatively-trained researchers (TMV, ESC, AF, ZBS) analyzed interview transcripts. We coded five initial transcripts synchronously to establish consensus and generate a codebook using NVivo software. We then coded remaining transcripts in pairs to ensure consensus throughout. The pair TMV and ESC coded 17 transcripts; AF and ZBS coded 18 transcripts as a pair. One transcript was coded by TMV alone and one was omitted from the coding process because the interviewee did not participate in the workshop. Individual team members (TMV, ESC, AF, ZBS, MW, LJ) identified themes from the codes and then met to establish consensus themes.

## Results

In total, 182 medical students and 70 ObGyn residents were invited to participate, of whom 24 medical students and 13 residents were ultimately interviewed (Figure 1). All five workshop facilitators were interviewed. Four central themes about the virtual experience were identified during data analysis: *the screen as a barrier*, *emotional safety, ease of access, and technology-specific features*. These themes are described further with supplemental exemplary quotes in Table 1.

**Table 1.** Supplemental quotes.

	Supplemental Quotes
Screen as a barrier *Participants noted obstacles to conversation and intimacy.*	R1. It does kind of make it a little bit difficult to have like a free flowing conversation because there are a lot of times where people will all try to kind of speak up at once, and then there’s a little bit of a delay for some people, and then you realize you’re accidentally talking or something when you didn’t even mean to, and then you both stop, and no one talks for like 30 seconds.
	R2. Different approaches to something that is polarizing like abortion can be seen through body language and like unspoken language and things like that which you kind of lose in a virtual sense. Like right now, because I’m trying to be poised I’m like playing with my sock, which is something that like I might do when I’m like nervous or thinking too much and you might not necessarily get that because I’m off screen.
	M1. I guess you can add that to the list of like bonuses for Zoom is that you really get to like watch all of these people and also watch people react, a little bit differently, because you have the checkerboard of like you know 40 faces or whatever it might be. And I thought that was a unique feature because I could also kind of prepare myself in case it was going to get more charged.
	R3. You can’t really have side conversations, it’s really just you know one person is talking and everyone else is listening, and I think it might have been interesting if it were in person to kind of have had the opportunity to talk with another co resident kind of one-on-one about different ideas.
	M2. I think it’s a lot easier to judge people when you’re not with them in the flesh…like when people are in person, it often humanizes other people’s responses more. [The computer screen is] like this shield and protection, and I think when we take that barrier down, it makes a lot easier for people to humanize each other and to be more real with those responses.
Emotional safety *Participants felt comfortable discussing sensitive topics.*	R4. Trying to put a little distance between you and ideas, to see what other people think about them, I think a virtual platform is actually really good for that…For some reason I felt like using zoom for that activity kind of diffused some of like the tension that could be there in my mind at least.
	F1. I think the virtual platform, maybe added a safety element, because you kind of feel like you’re in your own safe space where you’re not necessarily in the same space with other people that may have different opinions than you.
	M3. Especially with a sensitive topic, I think that there is a lot of power to having a safe space and to being in that safe space. … like you could always, you know, like turn your video off and take a breather, take a break, grab a sip of water or something. So it’s kind of nice to be able to have that opt out for a few minutes and then jump in if you did need it.
	M4. There’s some degree of protection that you feel when you’re just sitting in front of your computer screen rather than in front of a group of people…You wonder if your viewpoints will be incorrectly perceived by the group. So it feels a little bit safer to be behind your computer, especially in conversation setting that many of us aren’t as used to.
Ease of Access *The virtual format increased participants’ and facilitators’ access to the workshop.*	M5. If it was voluntary, and I had a choice between doing it over Zoom and having to go to a lecture hall, I may opt to do the one over Zoom and not actually walk to the one that’s going to physically take some time. I think it brings down some of those access barriers for people.
	F2. We had actually planned to drive to each other’s institutions so facilitate each other’s workshops, which now seems so quaint, like the concept of driving two hours to give one lecture, seems insane, but at the time we were like that’s normal, it’s not that far, and now the beauty of Zoom which weirdly I didn’t immediately recognize, is that we can facilitate each other’s sessions from home, like it’s super easy.
Technology-specific features *Zoom features streamlined aspects of the workshop and allowed for anonymous contributions to discussion.*	M6. We’ve all gotten so used to zoom especially you know as medical students, it’s something we do a lot. So that’s kind of nice to like just able to like instantly break out and talk and like come back and, like the way people can share their screens and stuff you know to like look at the survey things and talk at the same time that can be useful.
	F3. I do think, specifically for Four Corners, I think this works way better. Because in person, when you have people having to physically move around the room, it wastes a lot of time. Or even some of the other values clarification is where people stand on a spectrum. All of those where people are physically having to move, even though it’s more engaging, I find that it takes a lot longer. So I think in general, we are able to get through a lot more in a single hour virtually, than we are in person.

*Legend: M indicates a medical student quote, R indicates a resident, and F indicates a facilitator. Each number indicates a different interviewee*.

### The screen as a barrier

One disadvantage of the virtual format was that the computer screen created a barrier between participants, which was noted by a majority of the trainees (13 out of 24 medical students and 8 out of 13 residents) and all facilitators. Some participants noted that others kept their cameras off the entire session or stayed muted during the discussion, which they felt hindered conversation. Some felt that the flow of conversation was impeded over Zoom. They recounted instances when participants would accidentally interrupt one another due to delays in internet connection or because it was difficult to detect cues that another participant was about to speak (Table 1: R1), which might have been easier to distinguish in person.

In addition to maintaining the flow of conversation, participants felt that nonverbal cues were important for other reasons. Many trainees wished they could have seen other participants’ body language, such as signs of discomfort after the facilitator read a question aloud or during another participant’s response (Table 1: R2). On the other hand, one medical student did report feeling that some cues such as facial expressions were *easier* to detect over Zoom due to the ‘*checkerboard*’ of faces visible on the screen, making it possible to observe several individuals’ reactions at once (Table 1: M1).

One facilitator who also had experience facilitating in-person VCAT workshops described challenges due to the lack of nonverbal cues. They recounted difficulty ‘*read[ing] the room*,’ or gauging participants’ level of understanding and engagement, through the virtual format. They explained:

I had become pretty in depth with having a sense of the vibe of my room when I was doing this in person and making sure who I was losing because I could see their face really clearly. So if I could tell that one table just was not engaging, when we broke into small groups, I’d make sure that I went to that table and tried to engage them a little bit more… whereas here I can’t, I can’t see them, and I can’t try to bring them back in, so I think that’s probably my biggest [issue with the virtual format].

In addition to Zoom’s impact on group conversation, some participants also noticed the lack of individual side conversations over Zoom. They described wishing that they could process a thought with a peer sitting near them, rather than sharing all thoughts with the group (Table 1: R3). One medical student recalled texting a friend after the workshop to debrief, but expressed that they would have preferred to be able to discuss their thoughts in person ‘*like us standing in the hallway after*.’

Participants also felt that the workshop’s virtual format lacked intimacy. They felt their colleagues were more distant, less vulnerable, and hidden behind their screens. One resident reflected, ‘*[These are] impactful and important discussions to have, and I feel like if you just have it with a black screen it’s not as meaningful*.’ A few participants feared that this lack of intimacy may have prevented participants from humanizing one another’s responses to challenging and controversial questions (Table 1: M2). As one medical student explained:

I think that there’s some sense of anonymity in it being virtual even though you can see other people’s faces, you’re still behind a screen, and so I think that it can be harder to see other people as people rather than just their opinions.

While participants acknowledged the benefits of increased anonymity during a sensitive conversation, some trainees felt that they should be challenged to have uncomfortable conversations. Two students felt that the workshop may have been more valuable for medical trainees’ professional development if it had required in-person conversation, in order to better prepare them for future discussions or conflicts with healthcare colleagues and patients, ‘*propelling them into getting used to that scenario*.’

### Emotional safety

Although many felt that the virtual platform created barriers, approximately half of the participants (11 out of 24 medical students, 4 out of 13 residents, and 4 out of 5 facilitators) also expressed that the virtual platform created a sense of emotional safety (Table 1: R4, F1). Compared to an in-person classroom, trainees found that the anonymity provided by Zoom helped them feel more comfortable sharing their thoughts, at times referring to the virtual format as a *‘safe space*’ where they felt ‘*protect[ed]*’ (Table 1: M3, M4). One medical student explained,

It’s a little bit less personal, and I think maybe for some individuals with such a charged topic or such a, such a sensitive topic, maybe [virtual VCAT workshops] would be a little bit better. You just feel like it’s a little bit less putting yourself out there, less, less chance to feel embarrassed.

Another medical student felt that ‘*starting these conversations can be a little bit less intimidating over Zoom.’*

People also utilized the functionality of an online platform to increase their feelings of security. Some described how they hid uncontrolled facial expressions by turning off their camera or simply walked away from the computer when they needed an emotional break (Table 1: M3). One medical student appreciated being able to use the camera functionality to monitor and adjust their facial expressions:

You could have like a safe space, like you could turn your camera off if you want or just like not even be in view of it and be able to read these things and kind of formulate your thoughts, or you don’t have to see the reactions of your classmates as you’re reading stuff too. I think I feel like it kind of gave some safety to it.

Virtual classrooms also allow people to participate from a location of their choosing, often at home. Multiple facilitators felt that the comfort of participants’ home environments allowed them to talk more openly than they might in a different setting. As one medical student stated, ‘*People are just kind of inherently more comfortable when they’re in their own homes.’*

### Ease of access

The virtual format increased access to the workshop for participants and facilitators. Approximately one-quarter of those interviewed (5 out of 24 medical students, 2 out of 13 residents, and 4 out of 5 facilitators) mentioned that the virtual workshop was easy to access. Medical students liked being able to join the workshop from home or wherever was convenient (Table 1: M5). A medical student who was working nights during the workshop explained ‘*it would have been even harder*’ to participate if the workshop had been held in person. Trainees also described the ability to participate during rural or away rotations at a significant distance from their main campuses, when they otherwise might have been unable to participate. One resident explained:

From a logistics perspective, like residents at different sites can participate. Sometimes, with the didactics it’s hard to get everyone together, and so having people be able to kind of tune in from wherever they were I thought was helpful.

Facilitators also appreciated easier access to the virtual workshop. Facilitators discussed the benefits of being able to leverage the virtual platform to lead sessions at remote locations, either because their learners were at outlying sites or because they were asked to facilitate sessions at different institutions (Table 1: F2). Thinking of students rotating in remote locations, a facilitator reflected:

They don’t go to a lot of the teaching…because it was held in person, and they are hours away from here; and now, we can have them all attend to the same workshop, at the same time.

One facilitator felt the virtual option helped them secure an outside facilitator for their residents, which they thought might help their residents feel more comfortable:

Residents report to me, and I always felt like there was a power differential in terms of them expressing their opinions or ideas or beliefs, even if you attempt to be super supportive… And so I thought that that was a really interesting and innovative thing, that we could facilitate sessions for residents and medical students at remote locations. And then it just makes it so much easier.

Some facilitators proposed that this method of remote facilitation could allow the workshop to be expanded to other institutions where a family planning clinician may not be locally available or comfortable leading the workshop.

### Technology-specific features

Several participants, approximately one-fifth, described that the virtual workshop was efficient due to technological features (2 out of 24 medical students, 4 out of 13 residents, and 3 out of 5 facilitators). Participants and facilitators enjoyed quickly transitioning in and out of breakout groups and the ability to complete surveys ahead of time to maximize discussion time during the workshop (Table 1: M6). One resident described:

I liked that we were able to, one, sort of streamline breaking up into groups. I liked that, you know, all of the pre-work was completed online before, there wasn’t like a lot of clutter and I feel like it was something that was relatively compact in terms of its timing, but we accomplished a lot.

However, some people also described delays with the virtual format, such as slower internet speeds, which negatively affected the workshop’s efficiency.

Zoom-specific features also affected the virtual experience. Many participants liked the polling feature, as it allowed facilitators to easily present survey responses. Some appreciated the polls’ objectivity, displaying their colleagues’ anonymous survey responses, which many like this medical student saw as a true representation of their cohort’s beliefs:

A good amount of people believe certain things that you wouldn’t have expected, so I liked being able to see those numbers and put it into perspective.

A few participants also commented on the Zoom chat feature. One resident liked that participants could send a message to the facilitator if they didn’t want to share a thought or question aloud, ‘*if someone didn’t feel comfortable in that environment*.’ However, one facilitator described an experience during which they became the mediator between two anonymous participants expressing their thoughts in the chat:

It became sort of a back and forth of anonymous discussion that I was then reading to the group so, whereas in person, hopefully, those two people would have had the discussion with each other. Instead here, it became, you know, the anonymity allowed people to say things that they might not have been as comfortable saying in person.

Breakout rooms were another commonly utilized feature during the workshop. Some participants felt that the transitions in and out of breakout rooms were smooth and more efficient than if the transition to small groups had occurred in person. Those with previous in-person workshop experience also felt that the virtual platform saved time by decreasing the amount of time participants spent physically moving around the room (Table 1: F3). Some also felt that the virtual breakout rooms created more privacy and less ‘*chaos*’ compared to multiple small group discussions occurring at once, ‘*in a big room with a lot of noise it’s actually not a very fruitful conversation*.’

However, others felt that the transitions between online breakout rooms inhibited discussion among participants. A few participants expressed that more facilitators would be needed to effectively mediate conversations taking place in multiple breakout rooms. One medical student described being in a breakout room without a facilitator as ‘*awkward*.’ Facilitators echoed these sentiments. One facilitator felt that their sudden appearance in a virtual breakout room altered the course of conversation, and worried that if they closed the breakout rooms, they might unknowingly bring a robust discussion in a different room to an end prematurely; they summarized, *‘the virtual way of doing it really cuts off discussion.’*

## Discussion

Values clarification workshops on abortion have proven to be an effective tool for educating international healthcare workers about abortion and reducing abortion stigma [8,9]. They have also been shown to be an effective tool for ObGyn residents training at religiously-affiliated hospitals in the United States. ObGyn residents in this setting who participated in VCAT workshops showed increased acceptance of abortion care post-workshop [8]. Previous research has only evaluated the impact of in-person workshops, but there has not yet been an evaluation of a virtual adaptation of these workshops.

Many studies have evaluated the efficacy of online modalities for medical education and demonstrated similar efficacy to in-person learning, including students’ ability to retain knowledge and develop communication skills [11,12,14,15]. A separate qualitative analysis of this study’s data supported this hypothesis for VCAT in particular: participation in the virtual VCAT workshop helped trainees understand their own and others’ views on abortion and practice professional communication [16](Preprint), similar to outcomes for in-person VCAT workshops. Our study adds to the existing literature by describing how the virtual format affected participants’ experiences engaging in VCAT workshops.

Our findings suggest that using an online platform to deliver values clarification workshops on abortion provides both unique advantages and disadvantages to in-person instruction. The original workshop takes steps to create a safe environment for participants by having them participate using an anonymous colleague’s survey responses, rather than their own. Themes from the interviews we conducted with medical trainees highlight how the virtual platform additionally allowed for more comfortable discussion around a stigmatized topic. Participants attributed their feelings of emotional safety during these discussions to the distance between participants created by the virtual format. Additionally, the online format lowered barriers, primarily travel time and effort, to accessing the workshop for both trainees and facilitators.

One commonly cited drawback to online education is a lack of social connection among learners and educators. In medical education, learners have reported feeling less connected and described challenges to communicating virtually in online courses [17,18]. This finding is supported by data in our study. A majority of participants and facilitators in the virtual VCAT workshop described the virtual platform as a barrier to connecting with others, citing a lack of intimacy and difficulty detecting non-verbal communication cues. Educators should weigh how the virtual format may both contribute to emotional safety and simultaneously reduce intimacy among learners when determining whether a virtual format best fits the specific needs of their learners and learning environment.

Despite some limitations of online education, virtual VCAT workshops may be a timely intervention after the Supreme Court *Dobbs* decision. Recent data shows that 56 ObGyn residency programs, approximately one-fifth of all programs, are in states with the most restrictive abortion bans (Ryan Residency Training Program [6]. We cannot know how medical trainees’ attitudes and behavioral intentions towards abortion will be affected over time in the aftermath of *Dobbs*. In this context, virtual values clarification workshops may become increasingly useful for combatting abortion stigma, as remote facilitators of the online workshop can reach medical trainees in more restrictive states where trained facilitators may be unavailable or less comfortable leading workshops. The findings of our study may assist medical educators by helping them weigh the advantages and disadvantages of a virtual VCAT workshop in their particular legal and cultural context, providing evidence to inform whether this format may benefit their trainees.

Our study design is a strength of this research. This was a multi-institution study, and we recruited participants with a range of baseline attitudes towards abortion prior to the workshop as well as non-responders. Additionally, conducting semi-structured interviews allowed us to elicit participants’ nuanced descriptions of their experiences with the workshop, and produced a rich dataset. Despite efforts to recruit interviewees with a range of opinions about abortion, selection bias remains a limitation of our study. Additionally, we anticipate limitations to generalizability of our data, given that our cohort consisted of Midwestern medical trainees and faculty. As the workshop is expanded to other regions, future research should assess trainees’ experiences in other geographic areas.

Using a virtual platform to deliver values clarification workshops on abortion is feasible and provides specific advantages of anonymity, safety, and accessibility, although at the potential cost of reduced vulnerability among participants.

## Appendices Appendix 1. Voluntary baseline survey*

- What medical school do you attend?
- What is your age?
- What country were you born in?
- If you were born in the United States, which state were you born in?
- What specialty are you most interested in pursuing?

How much do you agree or disagree with the following statements? (Strongly Agree, Slightly Agree, Neither Agree nor Disagree, Slightly Disagree, Strongly Disagree)

1. The issue of abortion is of little importance to me.
2. I support the provision of family planning and contraceptive services.
3. I feel comfortable working to increase access to family planning and contraceptive services.
4. I support the provision of abortion services as permitted by law.
5. I feel comfortable working to increase access to abortion services as permitted by law.
6. I feel comfortable talking with my closest family members about my involvement with abortion care.
7. I would feel comfortable observing an abortion procedure.
8. I would feel comfortable performing or assisting an abortion procedure.
9. I am clear about my personal values concerning abortion.
10. I feel very conflicted about abortion.
11. I can clearly explain my personal values concerning abortion.
12. I can respectfully explain values concerning abortion that conflict with mine.
13. I feel empathy for women who have experienced abortion.
14. All women should have access to safe, comprehensive abortion care in the first trimester.
15. Access to first trimester abortion should be restricted to certain circumstances.
16. All women should have access to safe, comprehensive abortion care in the second trimester.
17. Access to second trimester abortion should be restricted to certain circumstances.

As a part of your career in the future, which of the following do you plan to do? (Yes/No)

1. Learn more about the need for safe, comprehensive abortion care.
2. Raise awareness about the need for safe, comprehensive abortion care.
3. Advocate making safe, comprehensive abortion care widely available.
4. Educate women about safe abortion services.
5. Refer women seeking abortion to safe services.
6. Provide or assist with safe, comprehensive abortion procedures.

- With which of the following genders to you identify? (Man, Woman, Non-binary, Transgender Man, Transgender Woman, Transgender Non-binary, Another gender)
- Do you identify with any particular religion or denomination? (Yes/No)
  - If yes:
    - How much do you incorporate your religious beliefs into all your dealings in life? (Not at all, A little, Somewhat, Quite a bit, A great deal)
    - How often do you attend a church, temple, mosque, or other religious meetings? (Never, Once a year or less, A few times a year, A few times a month, Once a week, More than once a week)

**The language used in this survey is from the original survey used in VCAT workshops. Institutions who utilize this survey may consider substituting more gender-inclusive language.*

## Appendix 2. Abortion statements survey for workshop participation

Please read the following statements and mark the answers that best reflect your personal beliefs. Please be honest.

1. Abortion services should be available to every person who wants them
2. People who have an abortion are ending a life
3. A person should be able to have an abortion even if their partner/spouse wants them to continue the pregnancy
4. Liberal abortion laws lead to more irresponsible sexual behavior
5. Young unmarried people should be allowed to have an abortion if they want one
6. Clinicians who specialize in ObGyn have a responsibility to perform abortions
7. Minors should be required to get their parents’ consent in order to have an abortion
8. A pregnant person who has a terminal disease should be counseled to terminate the pregnancy, even if it is desired
9. Most people do not seriously consider the consequences before having an abortion
10. People should be able to have a second trimester (13-24 weeks) abortion, if they need one
11. People who have second trimester abortions (13-24 weeks) are indecisive
12. People who have multiple abortions should be encouraged to undergo sterilization

## Appendix 3. Standardized interview guide

*Students*

- What medical school do you attend?
- Before the [date of session at that school] values clarification and transformation workshop session on abortion, had you ever participated in the values clarification and transformation workshop?
- Can you describe in a couple of sentences what the workshop was like for you?
- What was it like to hear your beliefs represented by others?
- What did you like about the session?
- What needs improvement?
- What did you like about using a virtual platform for this type of workshop?
- What didn’t you like about the virtual platform?
- Did you find yourself thinking differently about any of your beliefs after the workshop?
  - Do you remember which ones?
  - Was there a rationale that prompted you to think differently?
- Prior to the workshop, what was your general impression of the attitudes and beliefs about abortion held by the other students in your program?
  - Did this change after the workshop? If so, how?
- After participating in the workshop, how do you think your beliefs and your classmates’ beliefs about abortion affect societal stigma or acceptance of abortion?
- During the workshop, did you learn anything that surprised you about your classmates?
- What are *your* views on abortion?
  - Did your views change in any way as a result of the workshop?
- Were there any questions or discussions that made you uncomfortable?
  - Do you remember which one(s)?
- Will your experience in this workshop impact how you care for or counsel patients? If so, how?
- Any other comments or thoughts you’d like to share?

*Residents*

- What residency program are you in?
- What year in residency are you?
- Before the [date of session at that institution] values clarification and transformation session on abortion, had you ever participated in the values clarification and transformation workshop?
- Can you describe in a couple of sentences what the workshop was like for you?
- What was it like to hear your beliefs represented by others?
- What did you like about the session?
- What needs improvement?
- What did you like about using a virtual platform for this type of workshop?
- What didn’t you like about the virtual platform?
- Did you find yourself thinking differently about any of your beliefs after the workshop?
  - Do you remember which ones?
  - Was there a rationale that prompted you to think differently?
- Prior to the workshop, what was your general impression of the attitudes and beliefs about abortion held by the other residents in your program?
  - Did this change after the workshop? If so, how?
- After participating in the workshop, how do you think your beliefs and your co-residents’ beliefs about abortion affect societal stigma or acceptance of abortion?
- During the workshop, did you learn anything that surprised you about your co-residents?
- What are *your* views on abortion?
  - Did your views change in any way as a result of the workshop?
- Were there any questions or discussions that made you uncomfortable?
  - Do you remember which one(s)?
- Will your experience in this workshop impact how you care for or counsel patients? If so, how?
- Any other comments or thoughts you’d like to share?

*Facilitator*

- What institution do you work for?
- How long have you been in practice/out of residency?
- How long have you been a faculty at this institution?
- Can you tell me how you became involved with this project?
- Prior to your most recent experience facilitating a values clarification and transformation, or VCAT workshop, have you participated in or facilitated a VCAT workshop?
  - If yes, can you briefly share a little bit about that experience?
  - How did that prior experience compare to the current VCAT session?
- Have you led the Four Corners workshop with students, residents, or both?
- The start of this project overlapped with the COVID-19 outbreak and subsequent pandemic. Can you tell me how this impacted your experience developing and delivering the workshop?
- What did you think about using a virtual platform for this workshop?
  - What were some advantages?
  - Some disadvantages?
  - If you were advising someone else designing a similar values clarification project, would you suggest they have it virtually or in person? Why?
- Prior to the workshop, what was your general impression about the attitudes and beliefs about abortion held by the students at your institution?
  - (AND if they ALSO ran session with residents-the residents in your program?) Did this change after the workshop? If so, how?
- Were there any questions or discussions that made you uncomfortable?
  - Do you remember which one(s)?
- After participating in the workshop, how do you think your beliefs and those of your students and residents about abortion affect societal stigma or acceptance of abortion?
- What are *your* views on abortion?
  - Did your views change in any way as a result of the workshop?
- Any other comments/thoughts you’d like to share?
